# Forensic multidisciplinary involvement after terrorist attacks

**DOI:** 10.1080/20961790.2020.1820688

**Published:** 2020-11-02

**Authors:** Bertrand Ludes

**Affiliations:** Université de Paris, BABEL, CNRS, Paris, France

A terrorist attack is a sudden, unpredictable and unplanned action aimed to disrupt the infrastructure of the place where it occurs and causing large numbers of victims. Thus, the role of the public authorities is to rescue the victims as quickly as possible after securing the area and re-establishing the access routes. These operations are codified in action plans which are applied to the extent of structures and personnel not affected by the events and available to deal with the consequences of the concomitant or successive events.

Once rescue operations are finalised, the forensic investigation will start with two mains goals: identifying terrorists and victims.

A series of coordinated attacks occurred on 13 November in Paris, and the city's northern suburb, Saint-Denis where three suicide bombers struck outside the “Stade de France” during a football match. This was followed by mass shootings and a suicide bombing in five terraces of cafés and restaurants. In the meantime, three suicide bombers did a mass shooting in the Bataclan theatre and took hostages. These coordinated attacks resulted in 130 dead victims including 89 from the Bataclan.

The first responders and forensic teams were confronted with a variety of challenges which occurred as a result of the variability of the modus operandi of these events (shootings, hostage-taking, suicide-bombings), the condition of victim’s bodies (which ranged from complete to highly disrupted), the location of single and multiple scenes, both indoor and outdoor, and the fact that both national and international persons were affected.

It seemed interesting to us to present this subject from the angle of the lessons learned during the Paris attacks in November 2015, which proved to be decisive during the medicolegal operations after the Nice attack in July 2016.

The different articles of this special issue highlight the importance of quick identification of victims to establish the list of victims as soon as possible. To this perspective, it is proposed to differentiate disaster victim identification (DVI) operations from the medicolegal investigations to find the causes of death to look for foreign bodies related to the terrorist act and to assess the survival time for some victims. When DVI operations are performed in priority, rapid gather of primary and secondary identifiers is allowed and autopsies can be performed as soon as bodies left the identification table. The importance of postmortem (PM) imaging will be presented in this issue, in particular the PM computed tomography (PMCT) scanner which will not only indicate the causes of death but also show elements of identification, specify the location of foreign body and will constitute an important aid in odontological identification through dentascan images.

Once the living victims have been evacuated, the police forces present at the scene as well as the special anti-terrorism and identification units proceed to the removal of the bodies according to the victim identification protocol published by International Criminal Police Organization (INTERPOL). It is important that a forensic pathologist would be involved from the beginning of the operations on the scene to the transportation of the corpses to the medicolegal institute. Indeed, he/she will be able to assess the importance of the medicolegal investigations on the deceased and give some indications about the timeframe for carrying out these acts. He/she will make the link between the teams involved at the scene of crime and those of the forensic institute that will receive the bodies or any other structure that will be designated to perform the investigation to ensure the correct delivery of the bodies.

The article written by Arrighi and Charlot [1] is devoted to DVI techniques applied in the event of a terrorist attack. The different phases are described with an emphasis on the actions of the antemortem (AM) and PM teams and the holding of the conciliation commission which decides on the identification of the victims. These authors insist on taking care of the families of victims in a different place from that dedicated to the medicolegal acts. Presentations of the deceased to their families are made only after formal identification has been made with the assistance of clinical psychologists.

The article by de Jong et al. [2] presents PM imaging techniques and demonstrates the value of the PMCT. The diagnostic of vital lesions allows to specify the causes of death and to evaluate with caution as possible time of survival. The highlighting of identifying characteristics is also carried out by searching for prostheses or other implanted material (cardiac pacemaker, osteosynthesis material). These images are kept sealed and may be transmitted to experts in radiology at the request of the judicial authorities.

The articles by Delannoy et al. [3] and Tracqui et al. [4] retrace the forensic investigations performed after the terrorist attacks in Paris on November 2015.

The paper of Delannoy et al. [3] is about the cutaneous patterns of gunshot and secondary blast injuries after the events actions, where terrorists used weapons of war as well as explosive charges. These two modes of action can, when combined, create skin lesions with similar macroscopic appearances, which can sometimes go unnoticed due to body fragmentation. The authors show that the distinction of these lesions can be microscopic via the pathological study of the wounds but also clinical and depends on the size of the cutaneous lesions, along with their shape, number, and distribution on the body.

The article written by Tracqui et al. [4] shows that in case of ballistic injuries, death was most often obviously caused by craniocerebral injuries, extensive organ lacerations, and/or massive haemorrhage. Among the terrorists killed by bombing, the pattern of lesions included extreme organ lacerations and the presence of foreign bodies due to the shrapnel load (steel nuts, glass fragments) or the explosive charge fastening system of the devices. The discussion highlights the particular difficulties of interpretation encountered in the framework of ballistic injuries.

de Boer et al. [5] describe the procedure used in the case of fragmented bodies. In their opinion, it is essential to engage the services of forensic anthropologists for the precise study of bone lesions, especially in multiple artificial lesions caused by high-velocity projectiles, to aid in subsequent tissue reconstruction. This article aims to provide information and guidance to individuals involved in managing DVI operations with fragmented human remains. It outlines key issues that should be addressed during disaster preparedness planning and at the outset of an operation when incident-specific strategies are developed. It also addresses challenges during recovery and examination of fragmented remains, highlighting the importance of experienced specialists at the scene and in the mortuary. DNA sample selection and sampling techniques are addressed, emphasizing the need for rigorous quality control.

The odontological identification is presented in the article by Toupenay et al. [6]. The methods of analysis and identification by dentascan are presented as they allow a rapid and relevant comparison with the AM odontograms.

Soussy et al. [7] showed in their paper that forensic physicians were later in charge of medical forensic examinations of the surviving victims, in the medico-judicial unit of Paris, in the Hôtel-Dieu Hospital in the specific context of terrorist attacks. This article is of particular interest while few data are published in the medical literature about forensic management of these events, and they all concern PM forensic medicine. This article proposes a brief description of the organization of the medico-judicial unit of Hôtel-Dieu Hospital, Paris, before addressing the purposes and conduct of the medical forensic examination.

The paper proposed by Beauthier et al. [8] considers the different teams’ contributions (forensic pathology, anthropology and odontology, federal police and crime scene investigation) present both on the scene of the attack on the Brussels National Airport (Zaventem) and in the laboratory work (autopsies, sample studies). These authors still insisted on the major role concerning the technical and human difficulties of the AM team. The dangerousness of the PM team intervention is also highlighted in face of explosives or homemade explosive devices during the attacks in Brussels.

With all these contributions, we hope that this special issue will give a realistic overview of the multidisciplinary investigations of all the different forensic disciplines in action after a terrorist attack under the umbrella of special police forces and the magistrates.

Bertrand Ludes *Université de Paris, BABEL, CNRS, Paris, France* bertrand.ludes@parisdescartes.fr
